# Effect of the Elongational Flow on the Morphology and Properties of Polymer Systems: A Brief Review

**DOI:** 10.3390/polym13203529

**Published:** 2021-10-14

**Authors:** Rossella Arrigo, Giulio Malucelli, Francesco Paolo La Mantia

**Affiliations:** 1 Dipartimento di Scienza Applicata e Tecnologia, Politecnico di Torino, Viale Teresa Michel 5, 15121 Alessandria, Italy; rossella.arrigo@polito.it (R.A.); giulio.malucelli@polito.it (G.M.); 2 National Interuniversity Consortium of Materials Science and Technology, Via Giusti 9, 50121 Firenze, Italy; 3 Dipartimento di Ingegneria, Università di Palermo, Viale delle Scienze, 90128 Palermo, Italy

**Keywords:** elongational flow, elongational viscosity, film blowing, fiber spinning, strain hardening, morphology evolution, multiphase polymer systems

## Abstract

Polymer-processing operations with dominating elongational flow have a great relevance, especially in several relevant industrial applications. Film blowing, fiber spinning and foaming are some examples in which the polymer melt is subjected to elongational flow during processing. To gain a thorough knowledge of the material-processing behavior, the evaluation of the rheological properties of the polymers experiencing this kind of flow is fundamental. This paper reviews the main achievements regarding the processing-structure-properties relationships of polymer-based materials processed through different operations with dominating elongational flow. In particular, after a brief discussion on the theoretical features associated with the elongational flow and the differences with other flow regimes, the attention is focused on the rheological properties in elongation of the most industrially relevant polymers. Finally, the evolution of the morphology of homogeneous polymers, as well as of multiphase polymer-based systems, such as blends and micro- and nano-composites, subjected to the elongational flow is discussed, highlighting the potential and the unique characteristics of the processing operations based on elongation flow, as compared to their shear-dominated counterparts.

## 1. Introduction

A large number of processing operations for polymeric materials having high industrial relevance are dominated by elongational flow, i.e., a stretching deformation that, depending on the specific process, can be uniaxial or biaxial [1,2,3]. Fiber spinning, film blowing, blow molding and foaming, among a few others, are typical examples of processing techniques, in which the polymer melt is subjected to elongational flow [4,5,6]. Actually, elongational deformation also occurs in some processes essentially dominated by shear flow, such as extrusion and injection molding [7]; the variation of the flow channels and the entrance in the die section in extrusion, or the injection of the melt into the mold gate, represent some examples in which the melt is subjected to components of elongation flow [8]. Moreover, during the past few decades, plasticizing conveying methods and related devices (such as vane, eccentric rotors extruders and extensional flow mixers) based on elongational flow were continuously developed, demonstrating shorter processing times and higher mixing effectiveness as compared to conventional shear-flow-dominated techniques [9,10,11,12,13].

The great relevance of these processing operations justifies the interest in the study of the rheological behavior of polymer melts when subjected to elongational flow, documented by a massive number of scientific papers in the last 50 years. In fact, a thorough knowledge of the processing behavior of polymeric materials when subjected to the elongational flow is of fundamental importance for predicting the melt behavior and obtaining straightforward criteria that allow for the selection of the proper material, as well as the optimization of the processing conditions [14,15]. 

It is well established that the elongational rheological properties cannot be directly derived from those in shear [16]. More specifically, a correlation between the extensional behavior of polymer melts and the shear-viscosity behavior exists only in a linear range of deformations [17]. Since processing operations usually occur in the non-linear regime, it is obvious the importance of rheological measurements in elongation flow and the evaluation of the effect of external parameters (such as temperature, time and rate of deformation) and of the macromolecular architecture of the polymer on its elongational behavior [18,19]. 

Additionally, a characteristic feature of the elongational flow is its potential ability to modify the morphology of homogeneous polymer systems, through the occurrence of orientation phenomena of the macromolecular chains along the flow direction [20,21]. As a result, significant variations of the polymer microstructure and, thus, of its mechanical, optical or barrier properties can be obtained [22,23,24,25]. Interestingly, the ability of the elongational flow in inducing morphological evolution has been documented also for polymer-based materials showing a multiphase microstructure, such as polymer blends [26,27,28] and micro- and nano-composites [29,30,31]. In particular, the application of the elongational flow during the processing of polymer blends causes a deformation of the droplets of the dispersed phase, leading to a modification of the blend morphology with a remarkable influence on the material final properties [32,33]. Interestingly, in some cases, a kind of “compatibilizing effect” has been reported, since the results obtained for blends processed through the application of the elongational flow were very similar to those obtained with the traditional methods employed for improving the compatibility between the polymer components [34,35]. 

Similarly, it has been documented the effectiveness of the extensional flow in ameliorating the state of dispersion of micro- and nano-fillers in polymer-based composites. Moreover, especially for composite systems containing anisotropic fillers, the application of the elongational flow during processing brings about some preferential orientation of the embedded particles along the flow direction [36,37], with beneficial effects on the overall performances of the material. 

In this review, a brief overview on the main features associated with the application of the elongational flow during melt polymer processing is provided. First, the definitions and the theoretical basis associated with the elongational flow are discussed, while also considering the differences with the shear flow regime, both in isothermal and non-isothermal conditions; further, the main achievements regarding the rheological properties in elongation of the most industrially relevant polymers are critically reviewed, while paying particular attention to the correlations between the polymer macromolecular architecture and the rheological response. Finally, the effect of the elongational flow in modifying the microstructure and the morphology of homogeneous and multiphase polymer systems are elucidated, highlighting the processing–structure–properties relationships of polymeric-based materials processed through different operations with dominating elongational flow. 

## 2. Main Features of the Elongational Flow

**Figure 1:**
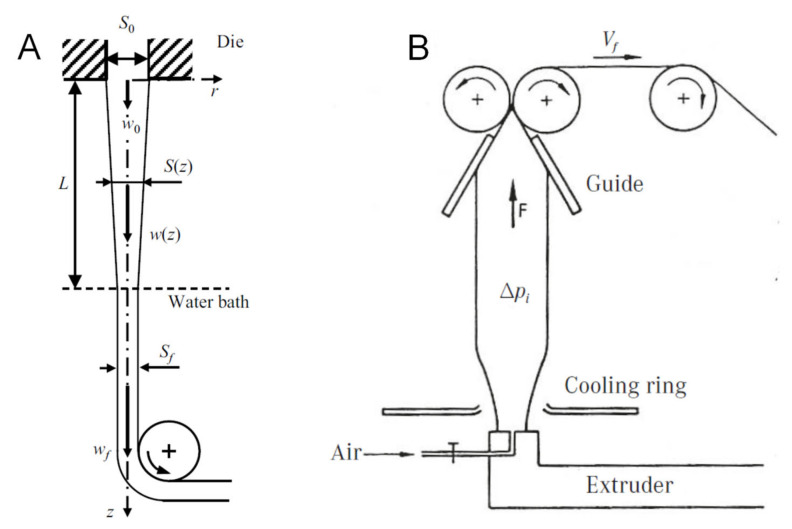
Schematic representation of melt-spinning (**A**) and film-blowing (**B**) processing.

The elongational (or extensional) flow is a particular kind of flow in which the velocity gradient develops in the same direction as the flow itself, rather than in orthogonal direction as in shear flow [38]. Two different types of elongational flow can be mainly distinguished, namely uniaxial and biaxial, depending on the number of stretching directions applied to a sample [39]. In particular, during uniaxial extension, the material is stretched in one direction, resulting in a size reduction in the other two directions. Differently, in biaxial elongational flow, the material is stretched along two different directions, with a corresponding size decrease in the third direction. The first type of elongational flow is encountered in melt-spinning operations (Figure 1A), while the polymer melt is subjected to biaxial elongational flow in film blowing (Figure 1B). 

Looking at the uniaxial elongational flow, the developed velocity profile can be described by Equations (1)–(3) [40]:

(1) $$v_{x}=\dot{\varepsilon }\cdot x$$

(2) $$v_{y}=-\frac{\dot{\varepsilon }}{2}\cdot y$$

(3) $$v_{z}=-\frac{\dot{\varepsilon }}{2}\cdot z$$

where *x* is the coordinate in the draw direction, and $\dot{\varepsilon }$ is the elongational or stretching rate, which can be a function of time and, in the case of uniaxial extensional flow, assumes positive values.

The stresses arising in uniaxial elongational flow are normal stresses, which can be calculated as reported in Equation (4):

(4) $$\sigma_{E}=\sigma_{zz}-\sigma_{yy}$$

The ratio between the elongational stress and the stretching rate is commonly defined as elongational or extensional viscosity, and it can be described through Equation (5):

(5) $$\eta_{el}=\frac{\sigma_{zz}-\sigma_{yy}}{\dot{\varepsilon }}$$

**Figure 2:**
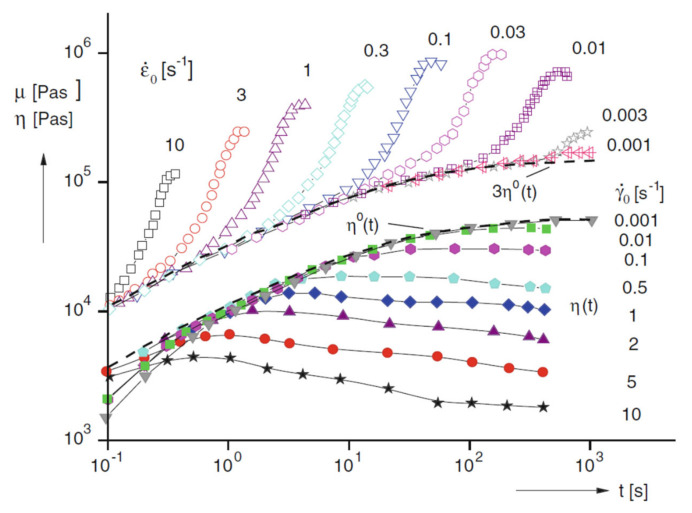
Isothermal shear and elongational viscosity as a function of time for an LDPE sample. Reprinted from Reference [41] under CC BY license.

Figure 2 compares the time dependence of isothermal shear with elongational viscosity for a low-density polyethylene (LDPE) sample [41]. In particular, the lower part of the figure shows the shear viscosity curves obtained through different measurements at various constant shear rates in a rotational rheometer. In the upper section of the figure, the elongational viscosity curves as a function of time are depicted, exhibiting a very different behavior as compared to that in shear. More specifically, the curves obtained at low elongational rate show an increasing trend as a function of time, up to a plateau; in these conditions, the material is in the linear range of deformation and the time-dependent elongational viscosity is three times higher than the time-dependent Newtonian shear viscosity. For the steady-state values, this relationship was first found by Trouton; therefore, it is commonly called the “Trouton law” [42]. As the elongational rate increases, a remarkable increment of the elongational viscosity values after a certain time interval can be observed. This phenomenon is called strain hardening and involves a progressively more pronounced resistance of the polymer melt to the extensional deformation. The occurrence of strain hardening has a beneficial effect on the regularity of the geometry of the sample during extension, hence ensuring a uniform deformation of the polymer melt in processing operations with dominating elongational flow [43,44].

**Figure 3:**
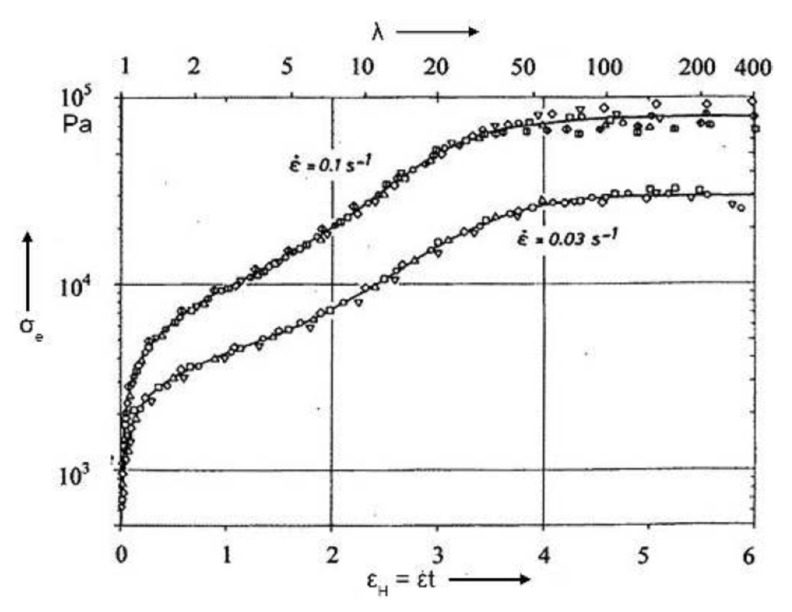
Tensile stress as a function of the Hencky strain at two elongational rates for an LDPE sample at 150 °C. Reprinted from Reference [41] under CC BY license.

The representation of the data coming from elongational measurements as elongational stress vs. Hencky strain ($\varepsilon_{H}$, defined as the natural logarithm of the stretching ratio) allows us to clarify the strain-hardening phenomenon. In fact, from the curves reported in Figure 3, an increasing trend of $\sigma_{E}$ as a function of the strain can be observed, highlighting a typical behavior of strain-hardening materials. It is worth noting that this representation of the elongational results allows us to magnify the plateau of the elongational stress for high $\varepsilon_{H}$ values, which is not evident in the curves reported in Figure 2. Though the stretching ratios required for reaching this plateau are much higher than those typically experienced by the polymers during the processing operations, the elongational measurements carried out at high strain are of particular interest for gaining fundamental insights into the deformation mechanisms of polymer macromolecules [44]. As a matter of fact, the existence of a plateau, a maximum or an overshoot of the elongational viscosity as a function of time or extension is still a matter of discussion in the literature [45,46,47]; this issue causes some uncertainties about the existence and the possible determination of a steady state in the elongational flow. In this context, Münsted et al. [48] claimed the fundamental role of the accuracy of the experiments and the uniformity of the sample deformation during the elongational tests, to avoid theoretical descriptions of effects based on experimental artifacts. In fact, from an experimental point of view, the achievement of reliable data from elongational measurements is rather complicated, and the availability of commercial advanced devices is limited. In contrast to the rheological properties in shear flow, for which the determination of the material-linear-viscoelastic behavior is quite straightforward, the elongational behavior is very difficult to evaluate, mainly due to the rapid and large deformation of the fluid elements, making challenging the generation of a regular elongational flow [49]. A further problem relies in the maintenance of the sample under extension for a sufficient time for the stress (in a strain-rate-controlled test) or strain (in a stress-controlled measurement) to reach a steady state, hence allowing for the determination of a steady elongational viscosity [48].

Among the different methods proposed for the evaluation of the elongational properties of polymer melts [50,51,52,53], the approach of Cogswell based on the converging or contraction flow analysis provides a simple way to estimate the trend of the elongational viscosity as a function of the elongation rate, through the evaluation of the pressure drop across the flow into an abrupt contraction [54,55]. Though this method involves several approximations, hence showing a limited usefulness, it has been widely exploited due to the easy generation of a converging flow of a polymer melt on a standard laboratory capillary rheometer. 

Following the analysis performed by Cogswell, the elongational viscosity can be expressed as reported in Equation (6):

(6) $$\eta_{el}=\frac{9}{32}{\left(n+1\right)}^{2}\frac{\Delta P_{ent}^{2}}{{\dot{\gamma }}^{2}\eta }$$

where $\Delta P_{ent}$ is the loss pressure due to the entrance effect, $\dot{\gamma }$ is the apparent shear rate, η is the shear viscosity and n the slope in the log–log flow curve plot [56]. Therefore, if the shear viscosity function of the polymer is known, the elongational viscosity can be derived from the measurement of the entrance pressure loss and the flow rate. As compared to other methods for the calculation of the elongational viscosity, the analysis of Cogswell provides relatively accurate results for both linear and branched polymers at high rates, while it fails at low rates [57]. 

As already stated at the beginning of the current paragraph, differently from the shear flow, in the elongational flow the velocity gradient is parallel to the flow of the material. This finding has a direct consequence on a fundamental characteristic of the elongational flow, i.e., its potential ability to induce a preferential orientation of the polymer macromolecules along the flow direction. In fact, when a polymer melt is subjected to a shear flow, the sample isotropy is preserved, notwithstanding the occurrence of anisotropic transients. Otherwise, during the application of the elongational flow, a stable orientation of elongated particles in the direction of the flow is achieved, resulting in the obtainment of anisotropic materials [58]. It is worth pointing out that these orientation phenomena occur in the melt portion of the material during a processing operation, in which elongational flow dominates; as an example, in the fiber-spinning process, although the final product is a solid filament, the orientation of the polymer macromolecules does not take place as a cold-drawing process, which may be performed afterwards as a separate processing step. 

Actually, in isothermal conditions, the orientation action provided by the elongational flow opposes to the relaxation phenomena occurring in the polymer melt due to the Brownian motion, which tend to randomize the material. Therefore, the achieved degree of stretching and orientation of the macromolecules is dependent on the balance of these two opposite events. More specifically, the following condition (Equation (7)) needs to be satisfied to obtain a substantial stretching of the polymer chains:

(7) $$\dot{\varepsilon }>\frac{0.5}{\tau }$$

where τ is the characteristic relaxation time of the polymer at specific conditions [40]. For entangled polymeric systems, for which the typical relaxation times are very high, moderate values of $\dot{\varepsilon }$ are required to obtain significant orientation levels, although some negative effects can derive from the decrease of the number of entanglements, and thus of the polymer relaxation time, during the processing carried out at high deformation rates. 

**Figure 4:**
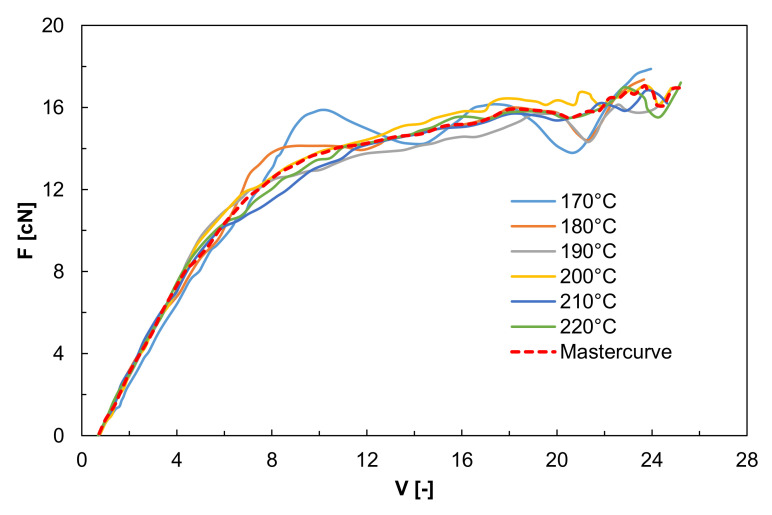
Rheotens curves for an LDPE sample obtained at different temperatures, and Rheotens-mastercurve. Adapted with permission from Reference [66]. Copyright (1996) WILEY-VCH Verlag GmbH & Co. KGaA.

Furthermore, if the application of the elongational flow is performed in non-isothermal conditions, the possible occurrence of crystallization phenomena during the extension needs to be considered, since factors related to the crystallization events may modify the existing orientation distribution. Furthermore, the presence of the orientation increases the crystallization rate [59,60]. As a matter of fact, in the common processing operations, in which the elongational flow is involved, such as fiber spinning or film blowing, the material is subjected to extension while the temperature progressively decreases. In this case, the polymer experiences non-isothermal elongational flow and the overall behavior of the material is determined by its rheological response, as well as by the variation of its rheological properties with temperature and by the rate of solidification (or of crystallization for semicrystalline polymers) [61]. The rheological behavior of a polymer melt subjected to non-isothermal elongational flow is typically evaluated through Rheotens (or other very similar devices) experiments, involving the application of a uniaxial extension under a constant tensile force on the molten filament [62,63]. During this kind of test, the tensile force needed for the elongation of the extruded filament is measured as a function of the draw ratio, obtaining tensile-force vs. drawdown-speed curves [64]. Rheotens test was extensively exploited for the characterization of the rheological behavior of polymer melts when subjected to non-isothermal elongational flow and for the modeling of many industrial-process operations, such as melt spinning or film casting, because of its ease of use and excellent reproducibility [65]. Furthermore, it has been proven that, for thermo-rheological simple polymer melts, the Rheotens measurements are invariant with respect to changes in testing temperature or in the average molar mass of the polymer, depending exclusively on the extrusion pressure (or the wall shear stress in the extrusion die) and the relative molar mass distribution of the polymer melt. This finding allowed the Rheotens-mastercurves (see curves reported in Figure 4) to be built that offer a direct and quantitative comparison of the deformability of polymer melts under uniaxial elongational flow at the typical operative conditions of industrial processes [66]. 

**Figure 5:**
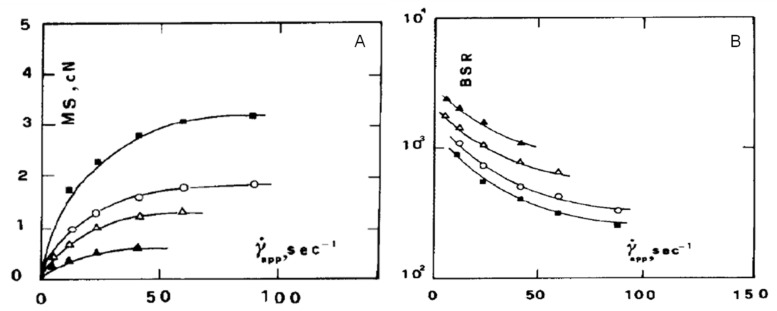
Melt strength (**A**) and breaking stretching ratio (**B**) as a function of shear rate for linear low-density polyethylene samples with different molecular weights. Reprinted with permission from Reference [67]. Copyright (1985) The Society of Rheology.

Obviously, during this measurement, it is possible to evaluate the force acting on the filament at breaking, the so-called “melt strength” (MS), and the ratio between the drawing speed at break and the extrusion velocity, corresponding to the “breaking stretching ratio” (BSR). The typical trends of MS and BSR as a function of the shear rate are reported in Figure 5. It is evident that a polymer can be easily processed through operations with dominating non-isothermal elongational flow when it shows high values of MS for sustaining the mechanical stress developed during the process and high deformability (high BSR values) for achieving the desired thickness [67]. 

## 3. Rheological Properties in Elongational Flow

In this section, the main achievements regarding the rheological properties of the most industrially relevant polymeric materials are reviewed, subdividing the discussion for results obtained in isothermal and non-isothermal elongational flow. Apart from the description of the typical rheological response of polymer melts when subjected to this kind of flow, particular attention is devoted to the influence of the macromolecular architecture of the polymer on its rheological characteristics and on the importance of the rheological properties in elongation on the material processability. 

### 3.1. Rheological Properties in Isothermal Elongational Flow

The rheological behavior in isothermal elongational flow has been widely studied especially for polymeric materials with a great industrial relevance, such as polyethylene (both high- and low-density), polypropylene and, in the recent years, polylactic acid and other biopolymers that attempt replacing traditional fossil fuel-based thermoplastics in many industrial applications.

From a general point of view, it is well established that the content of long-chain branching has a remarkable effect on the elongational behavior of the polymer melt, leading to the occurrence of a strain-hardening behavior [68,69]. An interesting study by Wolff et al. [70] on the elongational properties of a commercial low-density polyethylene (LDPE) demonstrated that, in the linear regime of deformation, the Trouton law was fulfilled, while, in the nonlinear regime, the presence of a large amount of long-chain branching induced the occurrence of strain hardening, with a rapid increase of the elongational viscosity as a function of the stress. 

**Figure 6:**
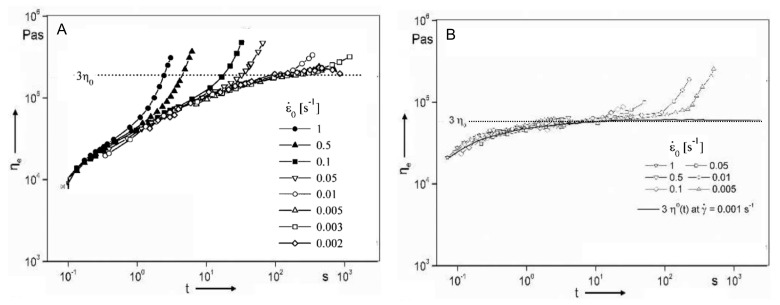
Temporal evolution of the elongational viscosities for (**A**) LDPE and (**B**) LLDPE samples at 150 °C and different elongational rates. Reprinted from Reference [41] under CC BY license.

Münstedt et al. [71] reported an unusual behavior for a series of elongational measurements performed on a conventional long-chain branched LDPE and a linear low-density polyethylene (LLDPE) sample. As observable from the curves displayed in Figure 6A, LDPE exhibited the expected strain-hardening behavior at high strain rates. The elongational behavior of LLDPE (depicted in Figure 6B) is quite surprising, since an unexpected increase of the elongational viscosity was recorded for low strain rate values. In other words, for LLDPE the strain hardening is more pronounced at low rather than at high strain rates. The authors provided a tentative explanation of this behavior considering the phase-separate microstructure resulting from the molecular structure of LLDPE that involves the coexistence of immiscible linear macromolecules and short-chain branched chains. Typically, for a homogeneous melt, the macromolecules form an entanglement network that is deformed by the cooperative motions of all chains; if the melt is deformed at high strain rates, the stress increases, while at low strain rates, a disentanglement mechanism prevails, resulting in a smaller stress rise. In LLDPE, the high-molecular-mass portion does not interact with the other macromolecules; therefore, only independent movements are permitted. 

As already discussed, the strain-hardening behavior is a key characteristic for the effective processing of the polymer melts through film blowing. Minoshima et al. [72] investigated the elongational properties of a variety of LDPE, high-density polyethylene (HDPE) and LLDPE, correlating the obtained results to the processing behavior of the polymers in melt spinning and film-blowing operations. They concluded that the highest stability during the film-blowing process was provided by LDPE samples, attributing this finding to the well-defined strain hardening exhibited by the materials. Furthermore, a significant effect of the molecular-weight distribution was documented, since HDPE and LLDPE samples with progressively narrow distribution showed an increased processing instability.

Aiming at improving the film-blowing processability of a linear HDPE sample, Münstedt et al. [73] formulated several LDPE/HDPE blends at different weight ratios. The assessment of the elongational behavior of the virgin polymers documented the expected strain-hardening phenomenon for the LDPE sample, with a progressive increase of the elongational viscosity as a function of the elongational rate; conversely, for HDPE, the strain hardening was not observed and the elongational viscosity exhibited a gradual decrease after a constant regime. The incorporation of 10 wt.% of LDPE into the linear polymer induced the appearance of a distinct strain hardening only at high elongational rates; although this behavior was not detected at low rates, the elongational viscosity values did not show a decreasing trend, suggesting the material uniformity during deformation. This result was confirmed by the calculation of the “inhomogeneity index” for the produced films; the lower values obtained for the blends-based blown films with respect to those of the virgin HDPE sample indicated the beneficial effect exerted by the presence of LDPE on the homogeneity and stability of the obtained materials. Similarly, La Mantia et al. [56] evaluated the elongational behavior of two types of HDPE/LDPE blends at different compositions and temperatures. In particular, the elongational viscosities of the investigated materials were calculated through the converging analysis by Cogswell. First, the results obtained for the virgin polymers confirmed the data already present in the literature on the same materials, confirming the reliability of the exploited calculation method. As far as the elongational properties of the blends are concerned, a significant influence of the Newtonian viscosity of the blend constituents was demonstrated, since for the system containing high viscosity LDPE a typical behavior of long-chain branched material was observed even for low content of HDPE. A further work by the same research group [74] investigates the elongational behavior of LDPE/LLDPE blends that have a high importance as far as the industrial applications is concerned, as these materials can combine the processability of LDPE with the good mechanical properties and the environmental stress cracking resistance of LLDPE. The obtained results in terms of elongational viscosity highlighted a strong strain-hardening behavior for the LDPE sample and the LDPE-rich blends; this characteristic progressively decreased with increasing the content of LLDPE and completely disappeared for virgin LLDPE, which showed a Troutonian behavior. Moreover, in this case, the presence of long-chain branching was invoked as responsible for the occurrence of the strain hardening. Furthermore, data obtained for blends containing LLDPE at different molecular weight pointed out a beneficial effect of high-molar-mass polymers on the elongational viscosity of the resulting materials. 

Apart from the content of long-chain branching, the molecular weight (M_W_) and its distribution have a remarkable influence on the polymer-elongational behavior. In this context, Münstedt et al. [75] evaluated the elongational viscosities of three LDPE samples differing for M_W_ and molecular-weight distribution. All the investigated samples exhibited similar behavior at low elongational stresses, and the values of their elongational viscosities coincided with the Trouton value. Conversely, in the high-stress region, the broadening of the molecular-weight distribution caused the achievement of higher viscosity values, with the appearance of a distinct maximum in the curves of steady-state elongational viscosity attributed to the presence of high-molecular-weight tails. Furthermore, the variation of the sample M_W_ caused a mere shift of the viscosity curves towards higher values, without modifying the curve trends. Stadler et al. [76] evaluated the elongational behavior of an LDPE sample synthesized in a laboratory-scale autoclave under high pressure, showing a high-molar-mass tail resulting in a bimodal molar-mass distribution and a lower content of long-chain branching compared to typical commercial LDPE. From the analysis of the time evolution of the elongational viscosity, the occurrence of a strain-hardening phenomenon was verified. This finding was ascribed to the long characteristic relaxation time of the fraction of high-molecular-weight macromolecules, demonstrating that the strain-hardening behavior can be properly modulated through the introduction of different contents of high-molar-mass components.

Another processing operation of great industrial importance dominated by the elongational flow is foaming. Stange et al. [77] investigated the influence of long-chain branching on the foaming behavior of blends based on linear and long-chain branched polypropylenes (PP). The assessment of the material rheological properties in elongation documented that the introduction of increasing amounts of branched PP caused the appearance of a progressively pronounced strain-hardening behavior, resulting in a continuous improvement of the blend-foaming behavior. In fact, a more uniform cell size distribution was found with increasing the content of long-chain branched PP and the blend containing 50 wt.% of this polymer exhibited the same foaming behavior as the virgin material. 

### 3.2. Rheological Properties in Non-Isothermal Elongational Flow

Most of the studies reported in the literature on the evaluation of the polymer rheological properties in elongational flow have been carried out in isothermal conditions, while less attention has been devoted to the rheological behavior of polymer melts experiencing non-isothermal elongational flow, notwithstanding its technological relevance for the assessment of the processing behavior of polymer melts. Similar to what was observed in the previous section, polyethylene- and polypropylene-based materials are the most investigated materials, given the widely exploitation of these polyolefins in processing operations, in which the non-isothermal elongational flow is prominent [78,79,80,81]. 

The first studies on the rheological response of polymer melts under non-isothermal elongational flow were performed by Denn et al. [82,83,84], aiming at assessing the influence of the melt elasticity on the stability of the melt-spinning process. The main achievements concerned the effect of the cooling on the increase of the initial rate of attenuation of the filament diameter and on the onset of the draw resonance instability. It was demonstrated that for viscoelastic materials, the decrease of the temperature during the spinning process caused a delay of the onset of processing instability, especially for long spin lines, while the melt is stabilized by the melt elasticity for short ones [85]. 

More recently, Muke et al. [86] evaluated the drawability of two PP samples differing for rheological and molecular characteristics through melt strength measurements. In particular, the influence of several extrusion and drawing parameters on the material extensional rheological properties was evaluated, showing that the polymer melt strength was remarkably affected by the extrusion rate. 

**Figure 7:**
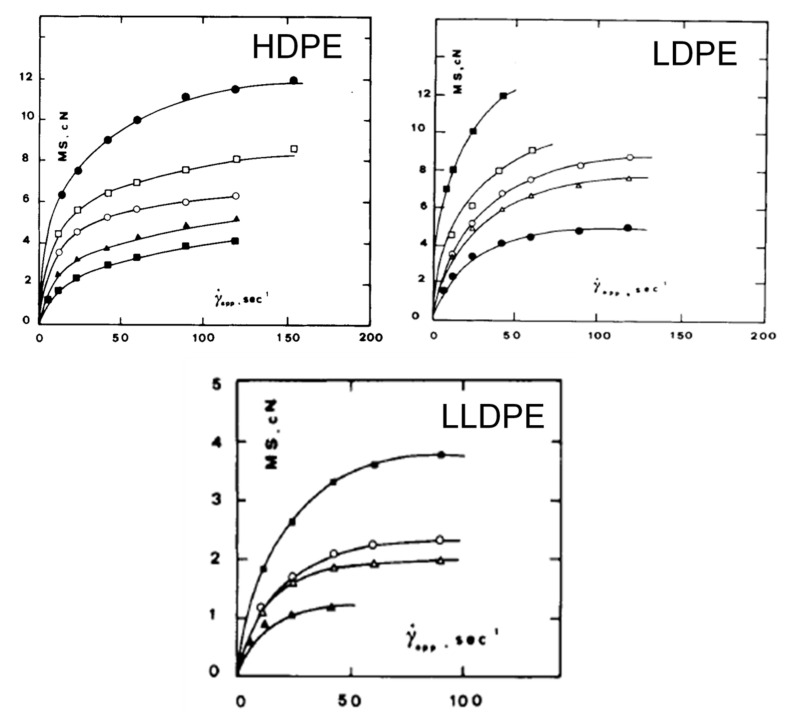
MS values as a function of the shear rate for HDPE, LDPE and LLDPE samples with different molecular weights. Reprinted with permission from Reference [88]. Copyright (1985) WILEY-VCH Verlag GmbH & Co. KGaA.

**Figure 8:**
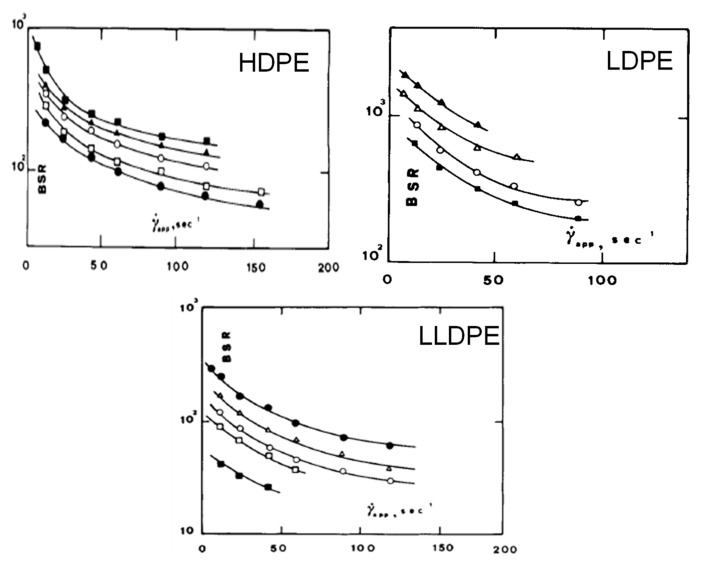
BSR values as a function of the shear rate for HDPE, LDPE and LLDPE samples with different molecular weights. Reprinted with permission from Reference [88]. Copyright (1985) WILEY-VCH Verlag GmbH & Co. KGaA.

As far as polyethylene-based materials are concerned, typically LDPE shows high values of MS and low deformability, while HDPE exhibits an opposite behavior [87]; the differences between the two materials can be ascribed to their dissimilar macromolecular architecture, pointing out the remarkable effect of the molecular weight and of the content of long-chain branching on the polymer rheological response. In this context, La Mantia et al. [88] compared the rheological behavior in non-isothermal elongational flow of a series of HDPE, LDPE and LLDPE samples with different molecular structure. As observable from the curves reported in Figure 7 and Figure 8, regardless of the polymer structure, MS showed a steep increase for low shear rate, approaching a plateau in the high shear-rate range. Conversely, BSR exhibited an opposite behavior, with a decreasing trend as a function of shear rate. This finding was explained considering the increased macromolecular orientation achieved by the material as a function of the shear rate (i.e., the extrusion velocity), which induced the attainment of a progressively more rigid and less stretchable filament. Concerning the influence of the macromolecular parameters, for all the investigated materials an increment of the molecular weight induced an increase of MS and a simultaneous decrease of the melt deformability. A peculiar behavior was documented for an LDPE sample obtained in a vessel reactor, showing a tree-like branching structure. In fact, for this sample, higher melt resistance and lower deformability were obtained, as compared to the expected values. This result was attributed to the presence of tree-like structures that hamper the mobility of the macromolecules, increasing MS and remarkably lowering the polymer stretchability. 

In the last decades, researches regarding the rheological behavior in non-isothermal elongational flow were mainly focused on polymer-based nanocomposites, given the increasing applications of blown films based on these materials in packaging industry. Particularly, polymer nanocomposites containing lamellar silicates have been the subject of a large number of studies; the general behavior reported for these materials involves the achievement of higher MS values as the nanoparticle content increases, due to the higher melt viscosity values provided by the introduction of increasing amounts of fillers [89,90]. Interestingly, the stretchability of the nanocomposite films is not, or only slightly, worsened as compared to that of the respective unfilled matrices; this finding is quite unexpected, as usually the presence of solid particles should induce a reduction of the melt deformability, which could compromise the filmability of these materials. In the case of lamellar silicate-containing nanocomposites, it has been demonstrated that, due to the very low dimension of the particles and the flexibility of the nanolayers, the embedded fillers do not act as defects, thereby not affecting the melt deformability [91]. Su et al. [92] investigated the non-isothermal elongational behavior of long-chain branched PP filled with different loadings of an organo-modified clays, reporting a strong influence of the filler content on the MS and drawability of the studied systems. In particular, it was observed a decrease of the MS values of the nanocomposites with increasing the filler content up to 6 phr, and a subsequent slightly decrease for a further increment of the clay loading; at the same time, the BSR of the materials exhibited a monotonic decrease as a function of the clay amount. The unusual trend of MS was associated with two concurrent events: (i) the possible degradation of the polymer matrix that occurs during the processing, causing a decrease of the matrix molecular weight and, hence, of the viscosity and melt strength; and (ii) the immobilization of the long-chain branches within the interlayer space, resulting from the achievement of intercalated hybrids, which minimized the effect of long chain branches. A similar behavior was also documented by McInerney et al. [93] for PP-talc composites; also in this case, the decrease of the MS of the filled systems was attributed to the degradation of the matrix during the processing step. 

## 4. Effect of Elongational Flow in Modifying Morphology and Mechanical Properties of Homogeneous Polymers

As discussed in the previous sections, a fundamental characteristic of the elongational flow is related to its ability to induce a preferential orientation of the polymer macromolecules along the flow direction, thus affecting the material morphology. Furthermore, for semicrystalline polymers, the application of the elongational flow influences the crystallization kinetics and the morphology and orientation of the formed crystalline structures [94,95]. In fact, the alignment of the polymer chains along the flow direction reduces the energy barrier for the crystallization, since the state of the polymer chains is closer to that of the final crystal when they are in oriented configuration rather than they are arranged in random coils [96]. 

All these structural evolutions induced by the application of the elongational flow have, in turn, a remarkable influence on the material final properties and, especially, on its mechanical performances [97,98]. In particular, as it is discussed in detail below, a very different behavior for semicrystalline and amorphous polymers has been reported. Briefly, for semicrystalline polymers, the application of an elongational flow usually results in an increase of the stiffness and a drastic decrease of the ductility as a function of the applied stretching [99,100,101,102]. Otherwise, for amorphous polymers, the elongation at break typically increases upon extension, and a kind of brittle-to-ductile transition is observed [103]. Therefore, the knowledge of the structure-process-properties relationships in processing operations involving the elongational flow is fundamental to predict the physical and mechanical properties of the final product, as well as to tailor the processing conditions and/or the polymer properties for achieving the envisaged performances.

As far as semicrystalline polymers are considered, it was demonstrated that the stable orientation of the macromolecular chains along the stretching direction achieved when these systems are subjected to elongational flow (especially in non-isothermal conditions) causes a progressive strengthening and stiffening of the material as a function of the draw ratio [104,105,106,107,108]. 

**Figure 9:**
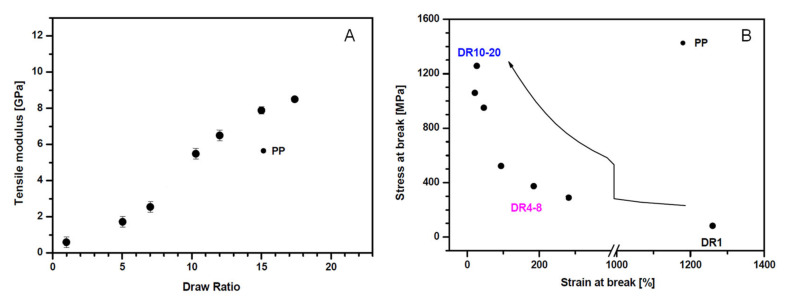
(**A**) Tensile modulus as a function of DR for melt-spun PP fibers, (**B**) stress at break as function of strain at break of selected PP fibers at different DR. Adapted from Reference [109] under CC BY 4.0 license.

This phenomenon is clearly observable in Figure 9A, which displays the trend of the tensile modulus as a function of DR for melt-spun PP fibers [109]. As the draw ratio increases, a severe increment of the fiber stiffness can be observed; furthermore, as documented by the results depicted in Figure 9B, a concurrent increment of the tensile stress and a drastic decrease of the fiber ductility verify as a consequence of drawing.

**Figure 10:**
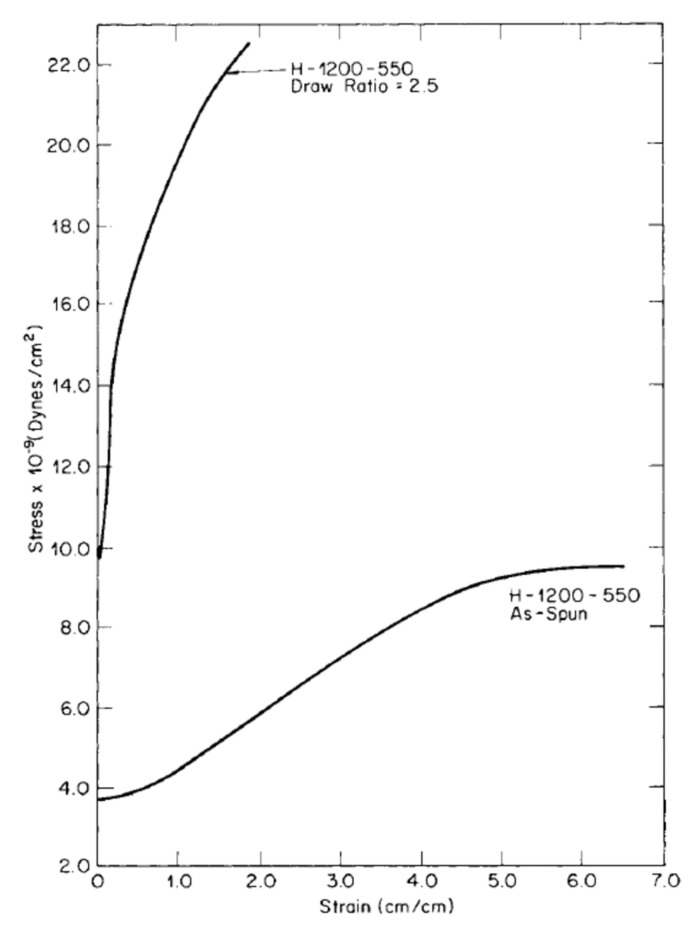
Stress-strain curves for as-spun spun and hot-drawn PP. Reprinted with permission from Reference [114]. Copyright (1978) WILEY-VCH Verlag GmbH & Co. KGaA.

Several studies have shown that the macromolecular orientation induces, in turn, an increase of the polymer crystallization rate and modification of the crystal structures, as well [110,111,112]. For instance, in the case of polypropylene-based materials, an elongation-induced evolution of the crystalline structure from a spherulitic to a cylindritic morphology was documented [113]. Furthermore, it was demonstrated that the observed development of the morphology is accompanied by remarkable modifications of the material mechanical properties, with the obtainment of gradually improved values of the elastic modulus and tensile strength as a function of the draw ratio, and a concurrent remarkable decrease of the elongation at break. In this context, Nadella et al. [114] evaluated the influence of cold-drawing (carried out at 25 °C), hot-drawing (140 °C) and annealing (140 °C) processes on the microstructure and mechanical behavior of a series of melt spun PP samples, documenting a correlation between the mechanical behavior and the birefringence of the obtained fibers. It was shown that the drawing process induced an increase of the chain orientation and a simultaneous disruption on the initial crystal structure, resulting in the achievement of extensive fibrillation that caused a drastic variation of the mechanical behavior of the fibers. In fact, as observable from the stress–strain curves of the melt spun and hot drawn fibers reported in Figure 10, the higher degree of chain orientation achieved in the drawn fibers, as compared to the as-spun filaments, led to the obtainment of improved elastic modulus and tensile strength values and decreased elongation at break, along with the disappearance of the apparent yield point.

**Figure 11:**
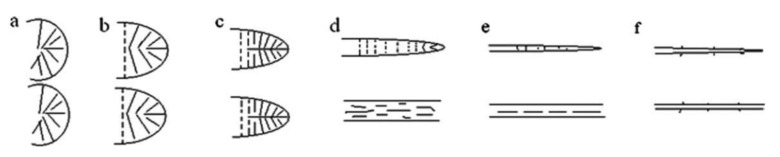
Schematic representation of the crystalline morphology evolution in PP blown films with the increase of DR. Reprinted with permission from Reference [121]. Copyright (2010) WILEY-VCH Verlag GmbH & Co. KGaA.

Furthermore, it was documented that, during a film-blowing process, the coupled effect of the biaxial elongational stresses that the polymer melt undergoes at the exit of the extruder die and the steep decrease of the temperature induces very complex orientation and morphology development phenomena [115,116,117]. In particular, for semicrystalline polymers, it has been shown that the stretching-induced crystallization phenomena are affected by the processing conditions, leading to the obtainment of different crystalline structures [118]. As an example, for polyethylene-based blown films processed at sufficiently high temperatures, the formation of shish-kebab structures, whose morphology and orientation depend on the level of stress imposed to the melt during processing, was frequently observed [119,120]. It is well-established that this peculiar crystalline arrangement originates from the orientation of the high-molecular-weight portion of macromolecular chains, which form fibrillar structures oriented along the film extrusion direction (machine direction, MD) during the processing. The formed fibrils act as nuclei for the bulk crystallization process, promoting the growth of crystalline lamellae in perpendicular direction (transverse direction, TD) with respect to the machine direction. Zhou et al. [121] documented the formation of shish-kebab structures in polypropylene-based blown films obtained at high DR values. In particular, as schematically depicted in Figure 11, at low DR values, a dominant spherulitic morphology was observed; as the DR increases, the spherulites were progressively deformed by the action of the elongational flow, until the formation of fiber-like structures oriented along the flow direction. A further increment of the DR induced the appearance of shish-kebab structures, which became dominant for DR > 100.

A quite recent work by Zhang et al. [122] documented the possibility to obtain shish-kebab-like crystalline structures also in PLA-based film subjected to an elongational flow for Hencky strain values above 2.0. It was shown that the formation of the shish-kebab morphology promoted a remarkable increase of the polymer crystallization kinetics, without negatively affect the global crystallinity content. 

Zhang et al. [123] investigated the structure–property relationships in LDPE, HDPE and LLDPE blown films obtained at different draw ratios. The results gathered from morphological characterizations showed remarkable differences in the crystalline morphology and orientation, which determined a different mechanical behavior. More specifically, HDPE films exhibited a strong anisotropic behavior at low DR, with higher tensile strength in MD than in TD. Conversely, the LDPE sample showed balanced mechanical properties at low DR and improved tensile strength in MD at high DR. Lastly, LLDPE films exhibited an isotropic behavior either for low or high DR values. The obtained results were correlated to the different effect of the applied elongational flow on the morphology of the three samples, as the anisotropy shown by HDPE and LDPE films was associated with the formation of fibrillar structures during processing, while this phenomenon was not observed for LLDPE. 

Quite recently, Zhao et al. [124] assessed the evolution of the microstructure and of the mechanical properties of blown films based on three polyethylene samples, namely a commercial LDPE, a metallocene-catalyzed ethylene–hexene copolymer and an ethylene–octene copolymer. It was shown that the elongational-induced crystallization phenomena occurring during the processing proceeded in a different way, depending on the polymer macromolecular structure, leading to the formation of a crystal-based network in LDPE film and a spherulite-like morphology in the other two samples. 

A self-reinforcing mechanism was reported by Čermák et al. [125] for different commercial grades of high-density polyethylene and isotactic polypropylene, processed in a twin-screw extruder equipped with a die containing a semihyperbolic convergent channel, thus allowing them to generate a high percentage of elongational flow in the melt. The morphological characterization pointed out the formation of fibrous structures in both materials; in particular, the obtained fibrils appeared mainly composed of elongated macromolecular chains, highly oriented along the extrusion direction. The achieved morphology promoted superior mechanical properties in a wide range of temperatures for the oriented materials with respect to isotropic samples, confirming the occurrence of a self-reinforcement mechanism induced by the application of the elongational flow. 

**Figure 12:**
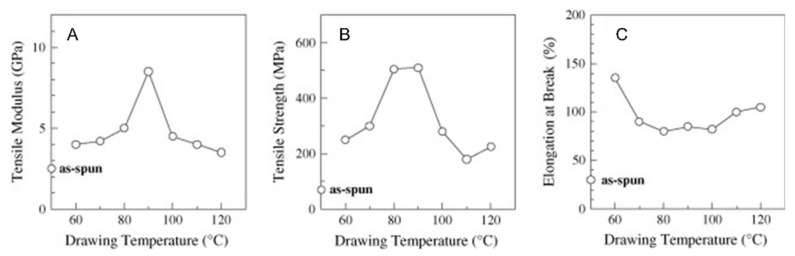
(**A**) Tensile modulus, (**B**) strength and (**C**) elongation at break of PLA fibers drawn at various temperatures. Reprinted with permission from Reference [126]. Copyright (2006) Elsevier.

An interesting relation between the mechanical properties of drawn PLA fibers and the drawing temperature was show by Furuhashi et al. [126]. The trend of the main mechanical properties as a function of drawing temperature (depicted in Figure 12) evidenced the achievement of a maximum for the tensile modulus and strength when the fibers were drawn at 90 °C; on the other hand, the elongation at break exhibited a decreasing trend as a function of the drawing temperature up to 90 °C, followed by a slight increase at higher temperatures. The observed behavior was correlated with results coming from structural analyses, which demonstrated an evolution from an oriented amorphous state to a highly oriented crystalline structure by increasing the drawing temperature up to 90 °C. A further increase of the drawing temperature caused a decrease of the crystallization rate and of the macromolecular orientation degree, because of the high achieved molecular motion, thus resulting in a rather brittle behavior. 

**Figure 13:**
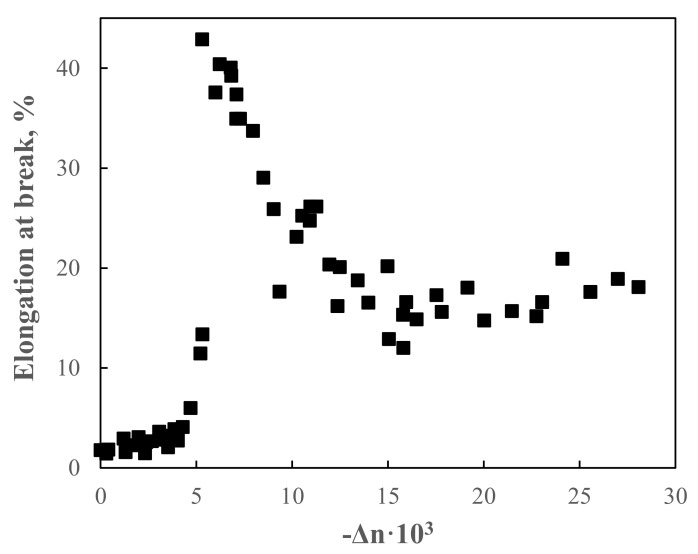
Elongation at break as a function of birefringence for a polystyrene filament. Data taken from Reference [103].

As briefly anticipated before, the scenario is completely different for amorphous polymers, for which an increase of the ductility upon elongation was widely observed [103,127,128,129,130,131,132]. Figure 13 shows the variation of the elongation at break as a function of the birefringence (which is indicative of the macromolecular orientation) obtained for polystyrene filaments subjected to a spinning-like experiment [103]. It is evident that the elongation at break shows an abruptly increase at a specific birefringence value of −6 × 10^−3^, after which a regular decrease is observed. Lastly, the elongation at break further increases at higher orientation values. This peculiar behavior, involving the occurrence of a sort of brittle-to-ductile transition, was ascribed to microstructural changes that occurred upon the application of the elongational flow; in particular, it was inferred that the more ordered morphology, involving oriented and stretched macromolecular chains, facilitates the macromolecular motion and improves the chain slippage. Furthermore, the compact arrangement of the parallel oriented and aligned polymer chains suppresses the formation and growth of cracks, bringing about to a ductile fracture [132].

## 5. Morphology Evolution of Polymer-Based Blends under Elongational Flow

During the past years, the continuous demand for innovative materials caused a steadily increasing interest towards the production of polymer-based blends, thanks to the possibility to achieve functional materials with tailored properties using polymer commodities. However, most polymers are thermodynamically immiscible in the molten state, due to unfavorable interactions between the components and the small gain in entropy resulting from the mixing of high-molecular-weight species [133]. As a consequence, polymer blends usually exhibit a heterogeneous morphology, which is characterized by the size and shape of the domains constituting the dispersed phase, as well as by their distribution in the matrix. Most commercial blends exhibit a typical droplet-like morphology, consisting in droplets of the dispersed phase embedded in the matrix, though fiber-like or lamellar structures can be also achieved [134]. Since the microstructure of a polymer blend has a predominant influence on the material final properties, the prediction and the control of the blend morphology are of fundamental importance for its end-use applications. 

The phase morphology of a polymer blend surely depends on the chemical nature of the components and on their ratio; additionally, due to the deformability of the minor phase embedded in the matrix, when processed in the melt, an in situ morphology can be developed [135]. Therefore, the kind of flow that polymers underwent during the melt processing operation greatly affects the mechanism of deformation of the dispersed phase, thus remarkably influencing the evolution of the blend morphology and, consequently, the material final properties. 

**Figure 14:**
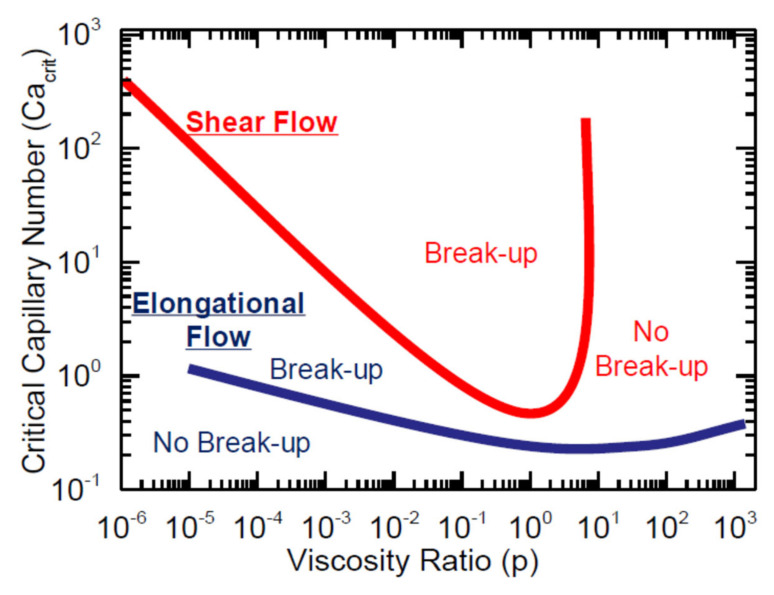
Critical capillary number as a function of the viscosity ratio in polymer blends according to the Grace’s analysis. Reprinted from Reference [143] under CC BY 4.0 license.

In particular, during processing, the droplets constituting the dispersed phase are continuously deformed and oriented and their final size and shape depend on the parameters controlling the deformation, as well as on the stability of the deformed particles [136,137]. In fact, depending on the processing conditions and on the physical properties of the blend constituents, each droplet can retain its original shape or can be deformed by the action of the applied flow. In this last case, the highly deformed particles of elongated shape can be unstable and further break up into satellite droplets. More specifically, the flow-induced evolution of the blend morphology is governed by two dimensionless numbers: the viscosity ratio of the blend (expressed as the ratio between the viscosity of the dispersed phase and that of the matrix) and the capillary number [138,139,140]. This last parameter represents the ratio between two counteracting stresses: the hydrodynamic stress, which is responsible for the elongation of the droplet, and the interfacial stress. As already discussed, during the flow the hydrodynamic forces tend to deform and to orient the droplets; the extent of this phenomenon depends on the viscosity ratio and on the effect of the interfacial forces, attempting to restore the initial particle shape. Moreover, the breaking up of the droplets may also occur if the capillary number is beyond a certain critical value [141]. Grace provided a systematic study about the variation of the critical capillary number as a function of the viscosity ratio in shear and elongational flow, demonstrating that the extensional flow is more effective than shear in deforming and breaking up the droplets [142]. In fact, as is observable in Figure 14, at a fixed viscosity ratio, a smaller value of critical capillary number is obtained in elongation than in shear; moreover, in contrast to shear flow, the elongational flow is able to induce the breakup of droplets at viscosity ratios higher than 4 [143]. 

The results obtained by Grace were confirmed for different polymer-based blends [144,145]; in particular, Utracki et al. [135] reported that the efficiency of the elongational flow in inducing morphology evolution dramatically increases for viscosity ratio beyond 3, leading to the deformation of the dispersed droplets to long fibrils. The possibility to obtain a droplet-to-fiber transition induced by the application of the elongational flow was demonstrated by several authors [146,147,148,149,150]. For instance, Chapleau et al. [151] reported the obtainment of a fibrillar microstructure for immiscible polycarbonate/polypropylene blends processed in a twin-screw extruder equipped with capillary dies of different lengths. The obtained results indicated that the elongational flow generated in the converging region of the capillary was effective in promoting a significant modification of the morphology of the formulated blends. Furthermore, morphological analyses confirmed, for the blends containing high amounts of dispersed phase, the formation of elongated fibrils, whose size was found to be remarkably affected by the viscosity ratio.

**Figure 15:**
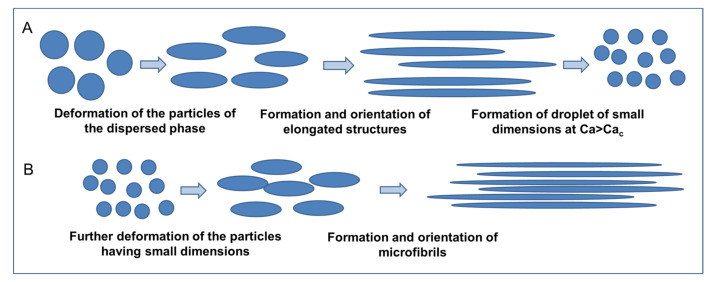
Schematic representation of the possible mechanism of the droplet-to-fibril transition in immiscible blends subjected to elongational flow: (**A**) Deformation of the original particles of the dispersed phase and (**B**) formation of microfibrillar structures.

Figure 15 schematically depicts a possible mechanism explaining the droplet-to-fibril transition observed in immiscible blends subjected to elongational flow. During the application of the extensional flow, the macromolecules constituting the matrix phase, similarly to a homogeneous system, tend to orient and align along the flow direction; at the same time, the particles of the dispersed phase deform, giving rise to the formation of elongated structures preferentially oriented in the stretching direction (Figure 15A). Once the critical capillary number is reached, the breakup of these elongated structures occurs, causing the formation of droplets of small dimensions that are further deformed by the action of the elongational flow, until the formation of microfibrils mainly oriented along the flow direction (Figure 15B). 

Fundamental studies on the mechanism of droplet deformation in uniaxial elongational flow were conducted by Delaby et al. [152,153,154], who found that the deformation of the droplets is lower than the macroscopic deformation of the material when the viscosity of the dispersed phase is lower than that of the matrix. These results were obtained through morphological observations of the dispersed phase structures, before and after stretching the melt, by freezing the sample morphology through a rapid quenching, without taking into consideration the possible shape retraction of the droplets during the quenching step [155]. This phenomenon was thoroughly evaluated by Gramespacher et al. [156] during uniaxial elongation measurements performed on immiscible polystyrene (PS)/polymethylmethacrylate (PMMA) blends. In particular, a two-step mechanism involving a viscoelastic recovery followed by the shape recovery of the deformed structures due to the action of the interfacial tension, was observed. Comparable results were achieved on similar PS/PMMA blends by Mechbal et al. [157], who described the double effect of the stress and the shape recovery resulting from the interfacial tension using the model proposed by Yu et al. [158].

Another fundamental factor influencing the development of the phase morphology during melt processing operation dominated by the elongational flow is coalescence; in fact, in the presence of intense interactions among the particles of dispersed phase, due to high concentration or restricted geometries, numerous collisions occur, hence possibly causing coalescence phenomena [159]. For instance, during melt spinning, the shrinking matrix compresses the droplets of dispersed phase, enabling them to migrate form the surface toward the center of the fiber and promoting coalescence events [160,161]. He et al. [162] reported the formation of a coalescence region during the melt spinning of immiscible polystyrene/polypropylene blends performed at take-up rates higher than 750 m/min, in which an increase of the diameter of the droplets of polystyrene along the spinning line was observed. 

**Figure 16:**
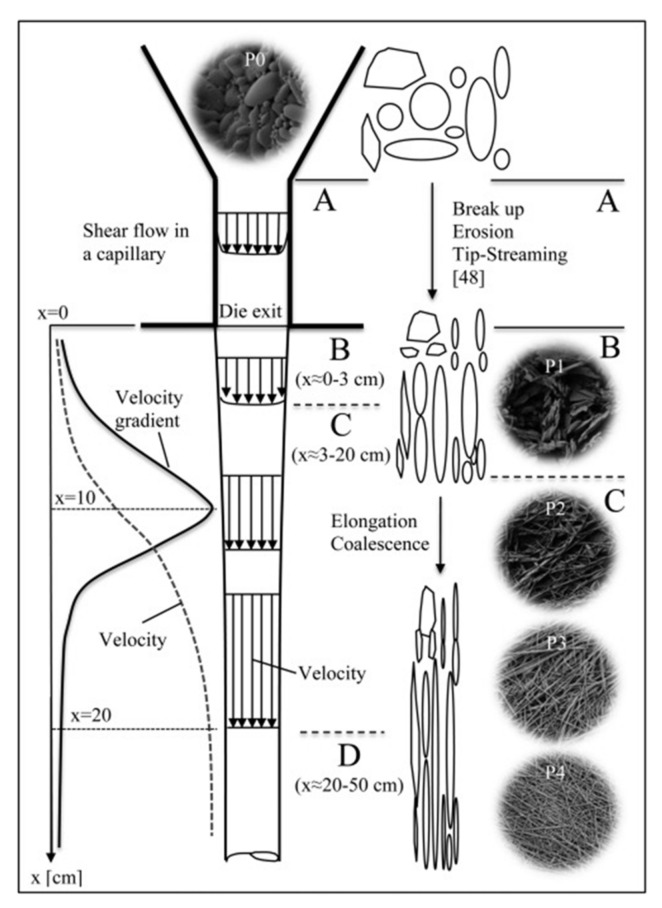
Mechanisms of fibrillation process along the spin line for a PLA filament. Reprinted with permission from Reference [167]. Copyright (2014) Elsevier.

Several studies concerning the morphology development of polymer blends during melt spinning focused on the variation of the fiber diameter, velocity, temperature and viscosity along the spinning line, highlighting the fundamental role of these parameters in the evolution of the dispersed phase from droplet-like to fibril-like morphology [163,164,165,166]. In this context, Tran et al. [167] investigated the morphology evolution of poly(vinyl alcohol) (PVA)/PLA blends during melt spinning, showing that the dispersed PLA particles evolved from rod-like structures to thin nanofibrils, because of concurrent stretching and coalescence. In particular, the morphological analysis of samples of the PVA/PLA blend filaments collected at different positions along the spinning line allowed them to explain the observed fibrillation process. As schematically depicted in Figure 16, a random morphology of the dispersed phase was observed at the exit of the capillary die, in which the dominant shear flow induced some stretching and breaking up of the particles, without causing fibrillation phenomena. Below the die exit, different events occur, namely the transition from shear to elongational flow and from a confined to a free-surface flow, the beginning of the cooling down of the fiber and the consequent non-uniform radial distribution of the viscosity on the cross-section of the filament. In the region, in which the velocity and tensile stress increase rapidly, PLA structures are deformed due to the stretching and oriented along the flow direction; furthermore, PLA particles tend to coalesce with adjacent dispersed phase to form continuous micro- and nano-fibrils. Finally, below the point, at which the filament reaches its glass transition temperature, the blend filaments were further stretched, with the achievement of PLA fibrils having a more elongated and uniform structure. 

**Figure 17:**
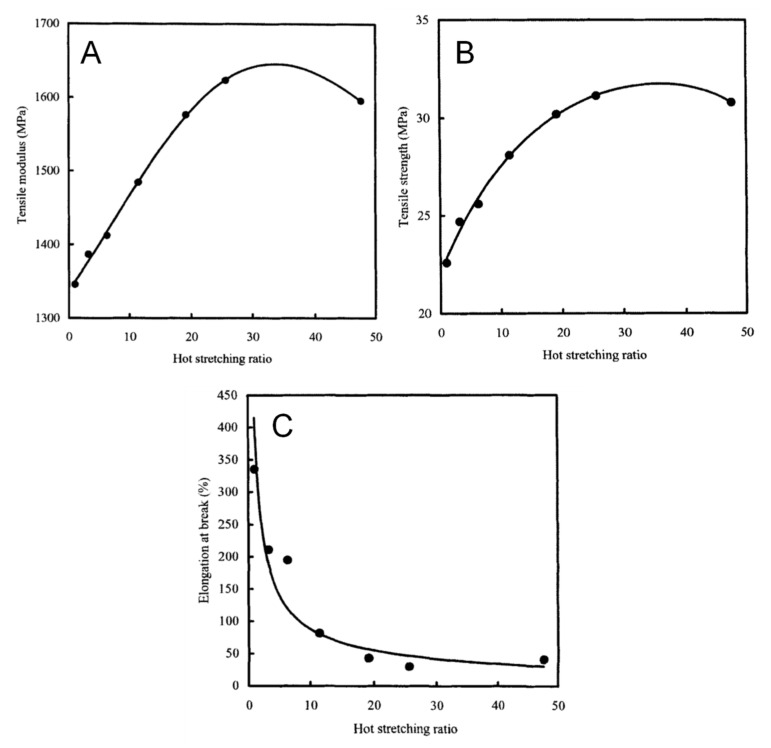
(**A**) Tensile modulus, (**B**) strength and (**C**) elongation at break as a function of the stretching ratio for a PET/PE blend. Reprinted with permission from Reference [172]. Copyright (2003) WILEY-VCH Verlag GmbH & Co. KGaA.

The main advantage of the evolution of the morphology of immiscible polymer blends experiencing elongational flow is the obtainment of anisotropic fibrillar structures, preferentially oriented along the flow direction that can improve the mechanical properties of the resulting materials, usually imparting higher stiffness and strength. Therefore, high-performance materials can be obtained in an easy and economical way, exploiting the processing operations dominated by the elongational flow typically employed for the industrial processing of polymeric materials. The general behavior reported in the literature for the mechanical behavior of immiscible polymer blends subjected to elongational flow during processing is very similar to that observed for semi-crystalline polymers (already discussed in the previous paragraph); typically, the formation of fibrillar particles and the increase of the degree of orientation of either dispersed or matrix phases bring about to an increase of the stiffness and strength as compared to the isotropic material, and a concurrent decrease of the ductility as a function of the intensity of the applied extension [168,169]. As an example, López-Barrón et al. [170] evaluated the morphology and the mechanical performances of blown films of immiscible polyamide 6 (PA6)/LDPE blends, documenting the formation of elongated PA6 fibrillar structures, mainly oriented along the machine direction, for blends containing low polyamide contents (10 wt.%) stretched at high draw ratios. Conversely, at high PA6 loadings (>30 wt.%), the minor phase formed lamellar structures that appeared deformed in both machine and transverse directions, though the deformation along MD was higher with respect to TD. The characterization of the mechanical properties of the blown films pointed out a remarkable influence of the intensity of the applied elongational flow, as the material showed, in MD, progressively improved tensile strength and decreased elongation at break with increasing the draw ratio. Differently, when the mechanical behavior of the blown films was characterized in TD, a decrease of the elastic modulus and of tensile strength was observed, without a specific trend as a function of DR. These results were explained considering the material anisotropy resulting from the preferential orientation of the deformed dispersed phase particles in the flow direction. Similar results in terms of mechanical properties were obtained by Morawiec et al. [171] for blends of recycled poly(ethylene terephthalate) (PET) and recycled HDPE processed through film blowing. In addition, in this case, the authors reported the achievement of higher elastic modulus and tensile strength for the oriented films along the machine direction, together with a concurrent decrease of the material ductility. Li et al. [172] evaluated the effect of the hot stretching on the mechanical properties of PET/PE blends in which, after the application of the elongational flow, the dispersed PET phase formed microfibrillar structures. As observable in Figure 17, a remarkable increase of the tensile modulus and strength was recorded for the stretched samples until a hot stretching value of about 35, after which the tensile properties remained almost unchanged. This behavior was explained by the authors considering that excessively high hot-stretching ratios can induce the breakup of the deformed PET particles, hence causing a decrease of the aspect ratio on the microfibrils. Looking at the results obtained for the elongation at break, a kind of ductile–brittle transition was observed at intermediate hot stretching ratios; this unexpected result was associated with the low deformability of PET fibrils at a high aspect ratio that possibly hindered the deformation of the matrix phase, thus preventing the achievement of high values of ultimate elongation at break.

**Figure 18:**
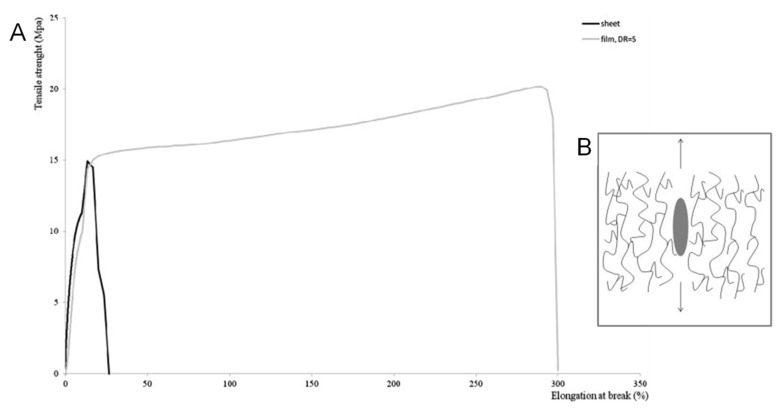
(**A**) Stress–strain curves of isotropic and anisotropic LDPE/PA6 samples and (**B**) sketch of the stress–strain test of the anisotropic film in MD. Reprinted with permission from Reference [34]. Copyright (2014) Elsevier.

An opposite behavior was observed by La Mantia et al. [34], who reported an orientation-induced brittle/ductile transition for LDPE/PA6 blown films. In particular, the mechanical behavior of the oriented films (measured in MD) was compared to that of an unoriented sample obtained through compression molding; the stress–strain curves reported in Figure 18A show a striking increase of the elongation at break for the anisotropic (oriented) sample as compared to the isotropic (unoriented) one. This unexpected result, similar to that commonly reported for oriented amorphous polymers, was explained considering the elongation-induced evolution of the blend morphology. In particular, as a result of the elongational flow applied during the film-blowing operation, the PA6 dispersed particles evolved from spherical droplets to rod-like elongated structures, thus increasing the interfacial area with the LDPE matrix with respect to the isotropic material and, consequently, improving the stress transfer between the two phases. Further, due to their preferential orientation along the same direction as the matrix macromolecules, the deformed particles did not act as defects during the deformation of the material (see the scheme reported in Figure 18B), hence allowing for the achievement of higher deformability. Interestingly, it was shown that the beneficial effect of the elongational flow in enhancing the ductility of LDPE/PA6 blends is very similar to that provided by the introduction of traditional compatibilizers (such as ethylene-glycidyl methacrylate), thus demonstrating a kind of “compatibilizing effect” of the elongational flow applied during processing [35].

Quite recently, a similar mechanical behavior, involving an elongational flow-induced brittle-to-ductile transition, was reported for a new class of all-thermoplastics based composites known as microfiber-reinforced polymer composites (MFCs). These materials, consisting of highly oriented microfibrils with high aspect ratio embedded in the matrix phase, are obtained from immiscible blends with a suitable viscosity ratio through an in situ fibrillation mechanism induced by the application of the elongational flow during processing [173,174,175]. MFCs have attracted a great interest in the last years, as they can provide an attractive alternative to traditional composites containing inorganic fibers, offering a solution to the problems of lightening to equivalent mechanical performances, while maintaining the recyclability of thermoplastics [176]. In particular, PLA-based MFCs have been widely investigated, since it has been demonstrated that the mechanism of in situ microfibrillation can simultaneously alleviate the brittleness, poor melt strength and slow crystallinity of this polymer [177,178,179]. Several authors reported an increased ductility of MFCs as compared to the unfilled matrix and/or to the native isotropic blend owing to the significant orientation of the high-aspect-ratio microfibrils, that allows an efficient stress transfer between the two phases [180,181]. 

## 6. Morphology Evolution of Polymer-Based Composites and Nanocomposites under Elongational Flow

Polymer micro- and nano-composites are an interesting class of materials for studying the effect of the elongational flow in promoting microstructural variations, since the state of dispersion, the size and, possibly, the orientation of the embedded nanoparticles can evolve upon the application of uniaxial or biaxial stretching. 

Despite the huge amount of scientific papers dealing with the formulation and the characterization of polymer-based micro- and nano-composites containing a large variety of fillers, only a relatively low number of studies concerning the evaluation of the elongation-induced structure development of these systems is present in the literature. At present, most of the investigations in this field regard polymer/clay nanocomposites, since (as it is thoroughly discussed below) they showed a peculiar behavior when subjected to elongational flow, which is completely different from that of other filled polymer-based systems containing different types of particles [182,183,184,185,186]. 

Some studies on composite materials containing glass fibers [187], graphite [188,189,190,191], halloysite nanotubes [192], hydrotalcites [193,194], cellulose nanocrystals [195], wood fibers [196] or carbon black [197] documented a beneficial effect of the application of elongational flow on the final morphology and properties of the materials. More specifically, upon uniaxial or biaxial elongation, a partial destruction of the filler agglomerates usually observed in composites obtained through melt mixing occurs; moreover, a preferential alignment of anisotropic filler and/or of particle clusters along the flow direction has been reported. Furthermore, it has been shown that all these morphological changes directly affect the final performances of the resulting materials; in general, superior stiffness, strength and optical and barrier properties are obtained for composite systems subjected to elongational flow, as compared to the isotropic counterparts. 

**Figure 19:**
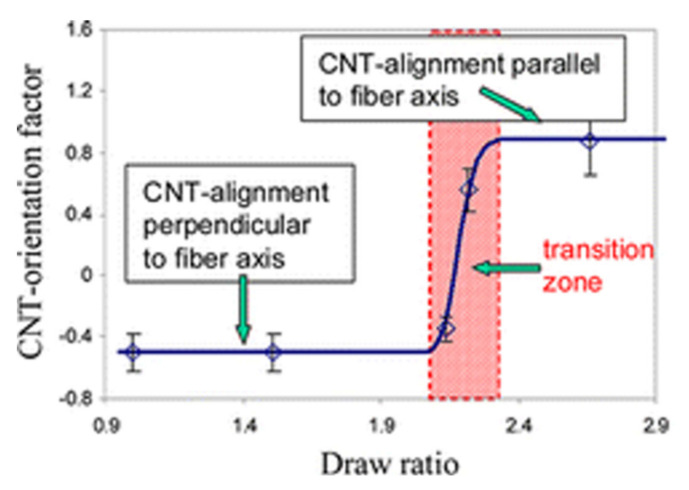
CNT orientation factor as a function of DR for PET-based composites. Reprinted with permission from Reference [204]. Copyright 2013 American Chemical Society.

A few works reported the effect of the elongational flow in inducing morphology evolution in carbon nanotubes (CNTs)-containing nanocomposites [198,199,200,201,202,203]. Generally, the application of the extensional flow during the processing causes the debundling of the CNT clusters formed during the melt mixing step because of attractive inter-tube interactions, resulting in an improvement of the extent of dispersion of the embedded fillers and in the obtainment of more uniform and homogeneous morphologies. Furthermore, a progressive alignment of CNTs along the flow direction as a function of stretching has been demonstrated. In this context, Vad et al. [204] evaluated the orientation of well-dispersed CNTs in PET-based nanocomposites fibers produced by melt spinning, showing a transition region in the filler alignment as a function of the draw ratio during the processing. As shown in Figure 19, the filler orientation factor (obtained through TEM observations) exhibited a S-shaped trend as a function of the draw ratio; in particular, at low draw ratio values, CNTs are mainly oriented in the orthogonal direction with respect to the fiber axis. For DR > 2, a transition from perpendicular to parallel alignment is observed, and the CNT orientation along the fiber direction is then maintained for higher DR. Furthermore, it has been demonstrated that this modification of CNT alignment during the melt-spinning process affects the structural properties of resulting materials, as the crystallinity of the filled fibers was found to significantly increase at high DR; further, the orientation of CNTs along the fiber axis, coupled with the high material crystallinity, caused the achievement of progressively higher values of tensile strength and lower ductility as a function of DR. 

The beneficial effect of elongational flow in improving the tensile properties of CNT-based nanocomposites has been widely documented [205,206,207,208]; in all these studies, the obtainment of enhanced stiffness and tensile strength has been associated to the increased polymer/CNTs interfacial area, resulting from the improved dispersion of the embedded fillers within the host matrices, which allows a more efficient stress transfer, as well as to the orientation of CNTs along the elongational flow direction. Furthermore, it has been demonstrated that the presence of highly oriented CNTs remarkably affects the crystalline structure of the polymeric matrices, which they are embedded in. In fact, aligned CNTs have been reported to act as nucleating agent for polymer crystallization, inducing in some cases the formation of shish-kebab structures that further contribute to enhance the tensile properties of the nanocomposites [209,210]. Moreover, oriented CNTs can act as template for the orientation of polymer chains, thus affecting the structure of the interfacial region. Some studies have documented that, due to the strong interactions established in the matrix/CNTs interfacial region, the polymer in the close proximity of the filler surface arranges in crystalline structures showing a more compact packing, a higher chain orientation and better mechanical properties than bulk polymer, thus improving the stress transfer between the CNTs and the polymer matrix [211].

However, the alignment of CNTs induced by the application of the elongational flow was found to exert a detrimental effect on the electrical conductivity of polymer/CNTs nanocomposites [212,213]. In fact, it has been shown that the increasing orientation of CNTs as a function of stretching significantly reduces the possibility for the fillers to form a conductive pathway through the matrix; in other words, the percolation network needed to ensure the electrical conductivity is disrupted as a consequence of the CNTs alignment and increasing amounts of CNTs are required for percolation as their degree of orientation increases. 

As briefly anticipated before, the most striking results in terms of elongation-induced morphology evolutions have been obtained for polymer nanocomposites containing layered silicates. Several studies documented a progressive orientation of the embedded clays as a function of the applied stretching, hence showing the already-described typical behavior of other polymer-based nanocomposites. In addition, it has been shown that the presence of small amounts of dispersed clays can facilitate the alignment of the matrix macromolecules, as the presence of well-dispersed platelets suppresses the relaxation of the polymer chains, thus promoting a high orientation degree [214]. 

Okamoto et al. [215] firstly reported the occurrence of orientation phenomena of the embedded nanoparticles under uniaxial extension in organo-modified clay (montmorillonite intercalated with stearylammonium ions)/PP nanocomposites showing an exfoliated morphology. Interestingly, for the samples stretched at low deformation rates, the authors reported the occurrence of some sort of reaggregation phenomena of the clay nanolayers, with the formation of “house of cards” structures. More specifically, during the application of the elongational flow, the individually dispersed clay layers are able to rearrange, giving rise to microflocculation events resulting from the electrostatic interactions between positively charged edges and negatively charged faces of the clay platelets. Similar results were obtained for a series of nanocomposites based on EVA and containing bentonite clay modified with cethyl-dimethyl-ethylammonium ions, whose morphology evolved from a dispersed state of fillers (presence of intercalated/exfoliated structures) to a less dispersed state (re-formation of clay tactoids) during the application of uniaxial elongational flow [216,217]. The observed reorganization of embedded clays upon elongation was explained considering that the stretching of the sample in one direction during uniaxial extension causes the contraction of the material in the other two directions, hence inducing a decrease of the cross-sectional area and, consequently, a reduction of the distance between clay platelets. Therefore, the decrease of the inter-platelet distance and the concurrent alignment of the macromolecular chains caused an evolution of the microstructure of the nanocomposites; furthermore, as the clay layers approach each other, the electrostatic interactions become stronger, causing the formation of flocculated structures. 

**Figure 20:**
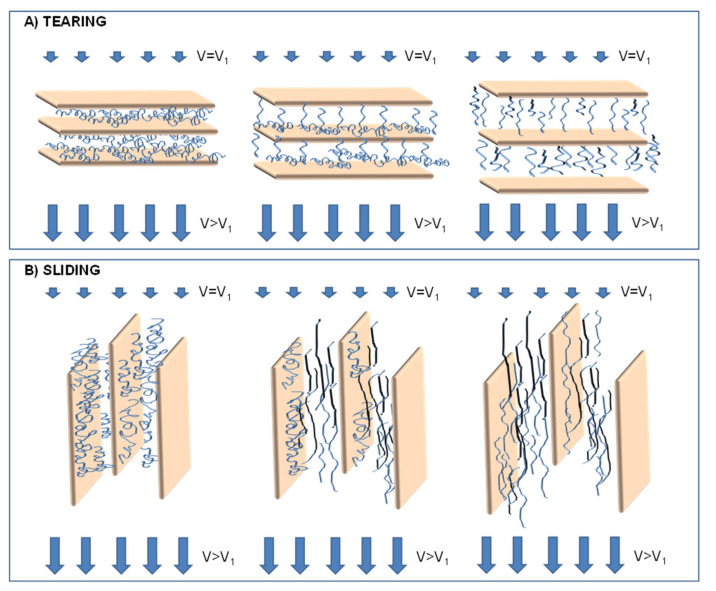
Proposed mechanisms (i.e., (**A**) tearing and (**B**) sliding) of elongational-flow-induced deformation of the tactoids in polymer/clay nanocomposites. Adapted with permission from Reference [218]. Copyright (2008) WILEY-VCH Verlag GmbH & Co. KGaA.

Different studies have demonstrated that the formation of “house of cards” arrangements is not the unique phenomenon occurring during the application of the elongational flow on polymer/clay nanocomposites; in fact, it has been widely reported that the microstructure evolution of these materials when subjected to extension is the result of different and concurrent mechanisms. In particular, La Mantia et al. [218] have documented a sort of elongational flow-induced transition from intercalated to exfoliated morphology in drawn fibers of PE-based nanocomposites containing a commercial montmorillonite modified with quaternary ammonium cations. In particular, the application of the elongational flow during the melt-spinning and, especially, during the drawing of the fibers promoted the increase of the interlayer distance in the tactoids and intercalated structures present in the as-compounded nanocomposites, giving rise to the formation of exfoliated morphologies or intercalated structures, characterized by a higher interlayer distance with respect to the isotropic materials. Moreover, in the drawn fibers, the dispersed clay platelets were preferentially oriented along the flow direction. The formation of exfoliated arrangements and the increase of the interlayer distance in intercalated structures upon extension has been explained considering that polymer/clay nanocomposites, when subjected to the elongational flow, show an intermediate behavior between that of a “traditional” composite material, containing non-deformable particles that can be oriented along the flow direction, and that of polymer blends, in which the dispersed phase can be deformed, broken and oriented by the action of the flow. In particular, different mechanisms (schematically shown in Figure 20) have been proposed to elucidate the elongational flow induced-deformation of the tactoids. In the scenario depicted in Figure 20A, due to the component of the velocity gradient along the flow direction, the clay layers are subjected to different velocities and some tearing of the particles may occur; moreover, the elongational stress resulting from the velocity gradient can further separate the nanolayers, hence increasing the interlayer distance. Differently, a sliding mechanism takes place when the clay platelets are oriented parallel to the flow (Figure 20B). In both cases, if the applied stress is high enough to exceed the electrostatic forces keeping together the clay platelets (which are already weakened by the presence of the intercalated polymer chains), individually dispersed layers are obtained, resulting in the formation of exfoliated structures. 

Several studies have further confirmed the achievement exfoliated or highly intercalated structures in polymer/clay nanocomposites subjected to elongational flow [219,220,221,222]. Undoubtedly, the final morphology achieved in these materials upon extension depends on the prevailing mechanism (i.e., flocculation of clays or improvement of intercalation/exfoliation). It has been shown that the formation of “house of cards” structures is favored when the elongational flow is applied in the molten state to nanocomposites already showing an exfoliated morphology; in fact, in the melt, the individually dispersed clay layers have sufficient mobility to rearrange, giving rise to the formation of flocs and clusters [218,223]. Furthermore, the polymer/clay interaction degree plays a fundamental role, as the achievement of a good adhesion between the two phases promotes the development of a “face-to-edge” morphology, causing clay rearrangements and flocculation [224]. Finally, it has been demonstrated that the amount of clay is a critical parameter in determining the prevailing mechanism, since an improved exfoliation upon extension was achieved for nanocomposites containing low particle content; on the contrary, filler aggregation phenomena are favored in the presence of high clay loadings, as this condition magnifies the electrostatic interactions among the particles [225].

**Figure 21:**
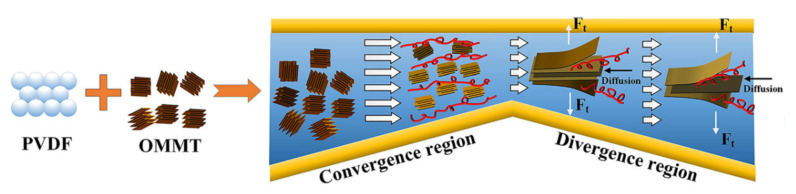
Schematic representation of debonding mechanism of exfoliated OMMT/PVDF nanocomposites in elongational flow field. Reprinted with permission from Reference [226]. Copyright (2021) WILEY-VCH Verlag GmbH & Co. KGaA.

A similar mechanism of clay exfoliation induced by the application of the elongational flow has been reported by Tong et al. [226] in polyvinylidene fluoride (PVDF)-based nanocomposites containing commercial organo-modified clays obtained in a vane mixer. In particular, as schematically illustrated in Figure 21, in the convergent region, the clay agglomerated are first broken to form stacks of tactoids, which experienced a “double layer peeling” process in the divergent region; moreover, the simultaneous orientation of the PVDF chains facilitates their diffusion within the inter-layer galleries, further inducing the clay exfoliation. This process is then repeated in the next convergent-divergent zone of the mixer, hence allowing for the gradual peeling of the clay nanoplatelets and the formation of exfoliated structures.

Obviously, all these observed morphological modifications induced by the application of the elongational flow cause a variation of the mechanical behavior of the nanocomposites as a function of the applied stretching. In particular, several studies demonstrated that the modification of the mechanical properties resulting from the application of the extensional flow is often associated to several factors, including (i) the evolution of the extent of dispersion of clay particles, (ii) their degree of orientation and (iii) the change of the crystalline structure and/or content of the polymer matrix resulting from the presence of oriented nanolayers [227,228,229]. Furthermore, different results in terms of mechanical properties were obtained depending on the prevailing mechanism of rearrangement of the nanocomposite microstructure upon elongation. Typically, a decrease of the material stiffness and strength as a function of stretching was documented for those systems in which the application of the elongational flow induces the formation of clay clusters [230], while improved tensile properties were recorded for nanocomposites, in which an improved dispersion and particle orientation were achieved as a result of the stretching [231]. Furthermore, similarly to what observed for amorphous polymers and some polymer blends, in some cases a kind of brittle-to-ductile transition was observed. This last behavior was generally reported for nanocomposites, in which the applied elongational flow promoted the achievement of exfoliated morphologies and a high orientation degree of the embedded particles, since the orientation of the individually dispersed clay platelets along the same direction as the polymer chains eliminates the stress concentration around the filler, hence facilitating the deformation of the material [232]. 

## 7. Conclusions 

Elongational flow is a particular kind of flow involved in many industrially relevant processing operations of thermoplastics, such as fiber spinning, film blowing, foaming and thermoforming. Aiming at assessing the processing behavior of polymers for such types of operations, the evaluation of the rheological properties of the materials that undergo this kind of flow is of fundamental importance; moreover, the knowledge of the elongational rheological behavior allows us to obtain simple and effective criteria for the selection of the proper material and the optimization of the processing conditions, as well. 

It has been widely reported that the application of elongational flow, especially in non-isothermal conditions, induces significant modification of the morphology of polymer-based systems, with the achievement of a stable orientation of elongated particles in the direction of the flow. Interestingly, elongation-induced orientation phenomena also occur in multiphasic polymer-based systems, leading to a remarkable variation of their microstructure and, thus, of their final performances (especially of their mechanical behavior).

The main achievements related to the effects of applying extension to homogeneous polymers and polymer-based multiphasic systems, such as blends and micro- or nano-composites, on the material morphology and mechanical properties can be summarized as follows:

(i) For homogeneous polymers, the orientation of the macromolecules and the subsequent modification of the material microstructure brings about different effects, depending on the semicrystalline or amorphous nature of the polymer. In more detail, semicrystalline polymers experience an increase of stiffening and strengthening upon stretching; meanwhile, for amorphous materials, a brittle-to-ductile transition is frequently observed.

(ii) or immiscible polymer blends, the application of extension results in a droplet-to-fibril transition, promoting the achievement of micro- or nano-fibrillar structures mainly oriented along the flow direction. In most cases, this morphology evolution causes an increase of the system stiffness and strength, even if in some cases a brittle-to-ductile transition quite similar to that observed for amorphous polymers has been reported.

(iii) For polymer-based composites, an improvement of the filler dispersion and a preferential orientation of anisotropic filler and/or of particle clusters along the flow direction is well documented, with a consequent enhancement of the material mechanical properties. A peculiar behavior is observed for nanoclay-containing composites, in which a sort of elongational-flow-induced transition from intercalated to exfoliated morphology occurs, due to the possibility to deform the dispersed clays that can be efficiently broken and oriented by the action of the elongational flow.

Finally, despite the significant amount of experimental research available in the literature about the effect of the application of elongational flow during processing on the morphology and properties of polymer-based systems, further studies would be useful to fully understand the correlations between the conditions imposed during the processing and the material final properties. Particular focus should be given to the assessment of these relationships in multiphasic polymer systems, aiming at achieving effective and simple methods for the prevision and control of their final morphology and, thus, performances. 

## Data Availability

The data presented in this study are available on request from the corresponding author.
